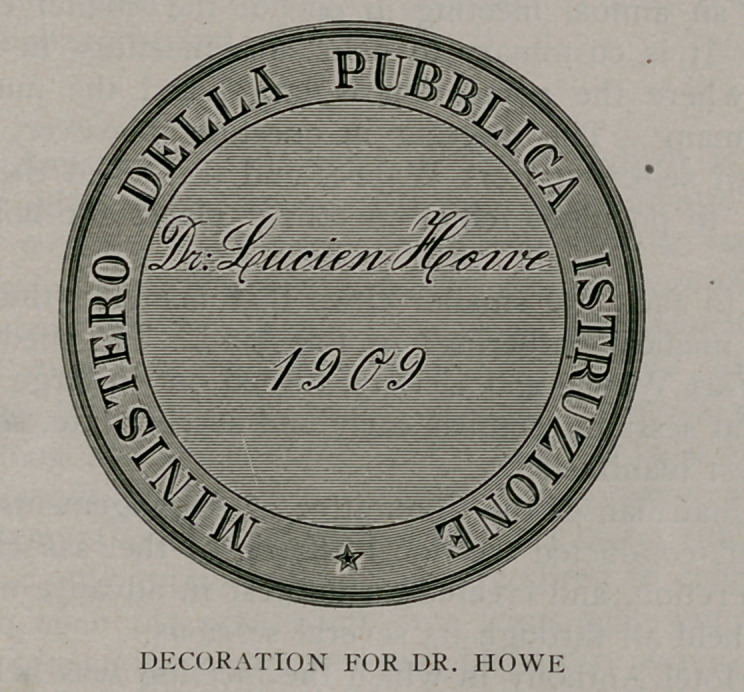# Doctor Lucien Howe Decorated

**Published:** 1909-11

**Authors:** 


**A Monthly Review of Medicine and Surgery.**

*EDITOR*

WILLIAM WARREN POTTER, M. D.

All communications, whether of a literary or business nature, books for review and exchanges, should be addressed to the editor, 238 DELAWARE AVENUE, BUFFALO, N.Y.

VOL. LXV. NOVEMBER, 1909. No. 4

### Doctor Lucien Howe Decorated.

THE Eleventh International Congress of Ophthalmology which was held at Naples last April, has proved to be an event of special interest. The attendance was unusually large over five hundred oculists from different parts of the world being

DECORATION FOR DR. HOWE

present, and the published transactions show the result of many original investigations. Among the most important of these is an article by Clausen of Berlin, concerning the identification of the germ of trachoma.

A unique feature of the congress was the announcement in advance that prizes, in the form of medals, would be given for the best and most important communication. One of these medals has recently been awarded to an American—Dr. Lucien Howe, of Buffalo. A copy of one face of the medal is here given. The other represents Victor Emanuel, the III. King of Italy.

The award was made for a paper on "The Measurement of the Lifting Power of the Adductors and of the Abductors." This investigation was undertaken in connection with a work in two volumes, on the muscles of the eye, recently published by the same author. By means of a simple appliance it has become possible to measure the actual strength of the muscles which turn an eye in or out, and thus decide in a given case of strabismus, the very important question whether to make a tenotomy of one muscle or the advancement of its opponent.

## Figures and Tables

**Figure f1:**